# True hermaphroditism with dysgerminoma: A case report

**DOI:** 10.1097/MD.0000000000020472

**Published:** 2020-05-29

**Authors:** Chun-Qiao Chen, Zheng Liu, Yu-Song Lu, Min Pan, Hui Huang

**Affiliations:** a The Department of Oncology, People's Hospital of Guilin; b The Department of Oncology, Fifth Clinical Medical College; c College of Medical Laboratory Science, Guilin Medical University, Guilin, Guangxi, China.

**Keywords:** chromosomal analysis, cryptorchidism, dysgerminoma, true hermaphroditism

## Abstract

**Introduction:**

True hermaphroditism is a rare and usually sporadic disorder. It is defined by the presence of both ovarian and testicular tissues together as ovotestis.

**Patient concerns:**

In this study, we reported a rare true hermaphroditism case with dysgerminoma. A 49-year-old woman developed masses in both inguinal regions for 30 years. Recently 3 months, the patient found that the size of mass in her left inguinal region was significantly increased.

**Diagnosis:**

After surgical resection, the results of immunohistochemical examination in left mass revealed a dysgerminoma with positive expression of placental alkaline phosphatase and octamer-binding transcription factor 3/4, and right mass was a cryptorchidism. Chromosomal analysis revealed the karyotype 46, XY. Combined immunohistochemical and karyotype analysis, a diagnosis of true hermaphroditism with dysgerminoma was made.

**Interventions:**

Radiotherapy combined with chemotherapy after tumor resection was used to improve her prognosis. Hormone replacement therapy with conjugated estrogen and medroxyprogesterone acetate were used to maintain her female characteristics.

**Outcomes:**

The patient underwent hormonal replacement and has been well for 6 months.

**Conclusion:**

The positive expression of placental alkaline phosphatase and octamer-binding transcription factor 3/4 could be 2 diagnosis markers of dysgerminoma. Surgery combined with radiotherapy and chemotherapy could improve the prognosis of dysgerminoma. Moreover, hormone replacement therapy with conjugated estrogen and medroxyprogesterone acetate was very helpful to maintain the female characteristic of patients with true hermaphroditism.

## 1 Introduction

In 1955, Dr Swyer first described true hermaphroditism in two 46, XY women with normal female external genitalia, hypoestrogenized vagina and cervix.^[1]^ More than half a century has passed, and over 200 cases of true hermaphroditism have been reported, but the pathogenesis at the molecular level remained unclear. True hermaphroditism is an extremely rare condition of disorder of sexual development. The karyotypes of patients with true hermaphroditism are mainly 46, XX, but many had 46, XY or a mosaic of 46, XX/46, XY.^[2]^ Patients with “true hermaphrodite” must have both mature ovarian and mature testicular tissue.^[3]^ It is a genetically heterogeneous condition. In this study, we found a 49-year-old woman with masses in both inguinal regions for 30 years. The clinical manifestations, immunohistochemically and cytogenetic examination were analyzed, and the diagnosis and treatment were discussed along with a literature search.

## 2 Case presentation

**Figure 1:**
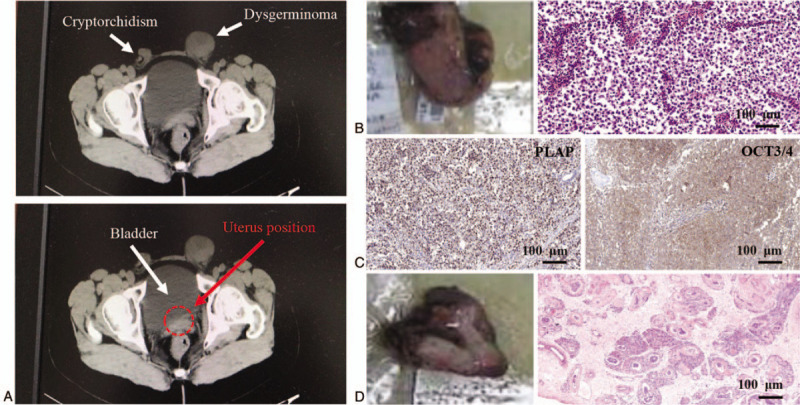
The results of computed tomography scan pictures and immunohistochemistry staining. A: The pelvic computed tomography scan picture of the patient. The white arrow indicates cryptorchidism, dysgerminoma, and bladder, respectively. The red arrow/red circle indicates the position of uterus, but the uterus is not seen. B: The surgically resected tumor specimen and its H&E staining. C: Immunohistochemistry staining shown the positive expression of placental alkaline phosphatase and octamer-binding transcription factor 3/4 in dysgerminoma specimen. D: The surgically resected cryptorchidism specimen and its H&E staining. PLAP = placental alkaline phosphatase, OCT 3/4 = octamer-binding transcription factor 3/4.

A 49-year-old woman was referred to the Oncology Department of our hospital in January 2019 because of masses in both inguinal regions for 30 years, with the increasing size of left mass recently. On physical examination, the patient presented with a 60 × 50 mm solid mass with clear borders in left groin areas and a 35 × 25 mm solid masses with clear borders in right groin areas. Both masses were no tenderness. On laboratory assessment, complete blood cells counts, urinalysis, renal and liver function tests, serum and urine electrolytes, and thyroid function tests were normal. Gynecological examination revealed the appearance of the external genitalia was female and of normal size. On a pelvic computed tomography scan (Fig. 1A), the uterus is absent (red arrow, right panel), and 2 masses in right and left inguinal region were seen (whit arrow, left panel), respectively.

**Figure 2:**
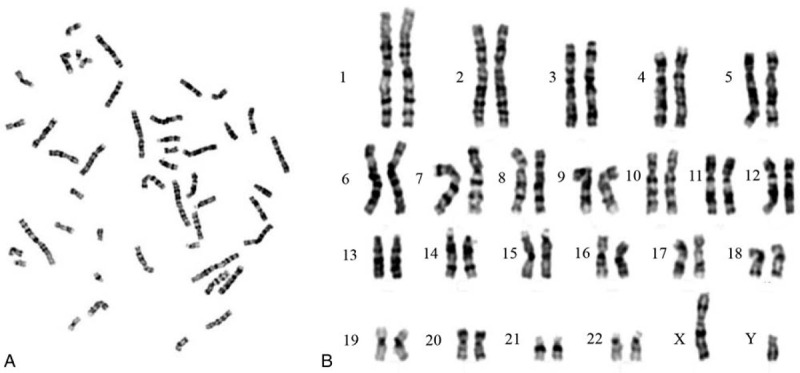
The diagnosis of patient with true hermaphroditism. Chromosomal analysis showed there was a 46, XY karyotype in A (dissociated chromosomes) and B (paired chromosomes).

In recent 3 months, the patient found that the size of mass in her left inguinal region was significantly increased. Due to an increased risk of malignancy, she underwent surgery to remove the mass in our hospital. The mass had reached 45 × 45 × 25 mm in size (Fig. 1B). During explorative laparotomy, there was no evidence of malignancy in the other pelvic organs. Pathology revealed the mass to be a dysgerminoma with the positive expression of placental alkaline phosphatase (PLAP) and octamer-binding transcription factor 3/4 (OCT 3/4) (Fig. 1C), while other markers including CD117, CD30, chromogranin A, synaptophysin, S100, Ki67 were all negative expression (Not shown). As the patient’ requirement, the right mass has been resected after 2 weeks and the histopathological examination revealed a degenerative testis and epididymis (Fig. 1D). To find out the presence of this anomaly, chromosomal analysis was performed and the result showed there was a 46, XY karyotype (Fig. 2). Based on the clinical and laboratory findings, the diagnosis of true hermaphroditism with ovarian dysgerminoma was confirmed. All procedures performed in this study involving human participants were in accordance with the ethical standards of the Guilin Medical University Committee and the Helsinki declaration of 1975, as revised in 2000. Written informed consent was obtained from the patient for the publication.

Since ovarian dysgerminoma originates from primordial germ cells of the ovary, it is highly sensitive to radiotherapy and chemotherapy. Thus, surgery combined with radiotherapy and chemotherapy was used for the patient for improving the prognosis. In terms of treatment, patients with true hermaphroditism must be treated as men or women based on factors such as stability of the sex gonads, reproductive tract, and external genitalia. Since the patient was genetically male due to her 46 XY karyotype, but socially and psychologically female in every respect, an important aspect of postoperative treatment was to maintain the female characteristics. Hormone replacement therapy with conjugated estrogen cream (0.625 mg daily) and medroxyprogesterone acetate (2.5 mg daily) was administered. At half-year outpatient follow-up, she had no evidence of relapse, with normalization of weight, development, and laboratory parameters. She underwent hormonal replacement and has been well for 6 months.

## 3 Discussion

True hermaphroditism is a rare condition and defined as the presence of ovarian and testicular tissue in the same individual, regardless of the patientʼs karyotype. Most of the patients with true hermaphroditism have female internal genital organs.^[4]^ The incidence rate is ∼1/100,000 live births and the degree of external genitalia varies between males and females.^[5]^ The testis and ovaries of patients with true hermaphroditism may be combined to form ovotestis, or they may exist separately. The presence of an ovotestis is the most common, followed by the presence of an ovary, whereas the presence of a testis is the least common.^[6]^ The patient in the present case was a 49-year-old woman with right cryptorchidism for over 30 years. Pathological examination revealed the presence of both types of gonads and the karyotype was 46 XY, which is a typical true hermaphroditism.

In the diagnosis of ovarian dysgerminoma, pathology and immunohistochemistry are of important reference value. PLAP and OCT 3/4 are currently recognized and widely used as tumor markers for the diagnosis of dysgerminoma, while OCT 3/4 expression is the most helpful in predicting risk of malignancy.^[7]^ PLAP is highly expressed in primate placental tissue, and sensitivity and specificity of PLAP for germinomas were 94% and 97%, respectively.^[8]^ OCT 3/4 is a transcription factor specifically expressed in mammalian totipotent embryonic stem and highly expressed in gernimomas.^[9]^ Combined with the clinical characteristics and histopathological examination, the diagnosis of ovarian dysgerminoma could be confirmed.

Until today, the etiology and pathogenesis of true hermaphroditism remains unclear, but sex chromosome abnormalities, abnormal gonadal development and related endocrine disorders during embryonic development may be implicated.^[2]^ Previous studies shown that sexual differentiation and gonadal development required the involvement of SRY gene on chromosome Yp11.2, NR0B1 gene on chromosome Xp21.3, NR5A1 gene on chromosome 9q33, CBX2 gene on chromosome 17q25, MAP3K1 gene on chromosome 5q11.2, DHH gene on chromosome 12q13, AKR1C2 gene on chromosome 10p15, ZFPM2 gene on chromosome 8q23, and SOX9 gene on chromosome 17q24.^[10–12]^ Male sexual determination is initiated by SRY, which activates a cascade of genes that lead the embryonic gonad to develop into a testis.^[13]^ If the patient's gonad is the testis, fetal testicular Sertoli cells then produce Müllerian inhibitory substance, which is responsible for the regression of the Müllerian ducts. At the same time, fetal testicular Leydig cells produce testosterone from cholesterol to generate activated dihydrotestosterone for subsequent differentiation of male external genitalia. If the gonad is the ovary, it does not produce the Müllerian inhibitory substance, and the Müllerian ducts would otherwise develop into the uterus, fallopian tubes, and cervix.^[14]^ In the present case, the patient lived as a woman, exhibited women secondary sexual characteristics, had no evident abnormalities in the external genitalia. This may indicate that the patient's androgen secretion was not sufficient to drive the development of the scrotum and penis due to the reasons of testosterone production deficiencies, dihydrotestosterone deficiency, androgen insensitivity, and defects in anti-Müllerian hormone or its receptor during the embryonic stage.

## 4 Conclusion

In this study, we reported a rare case of true hermaphroditism with dysgerminoma and the diagnosis along with treatment has been discussed. Our study suggested that PLAP and OCT 3/4 both could be the diagnosis makers of dysgerminoma. In the course of treatment, hormone replacement therapy with conjugated estrogen and medroxyprogesterone acetate could be used to maintain the female characteristic of patients with true hermaphroditism.

## Author contributions

**Conceptualization:** Chun-Qiao Chen, Zheng Liu, Hui Huang

**Investigation:** Chun-Qiao Chen, Yu-Song Lu, Min Pan

**Writing – original draft:** Chun-Qiao Chen, Zheng Liu, Hui Huang

**Writing – review & editing:** Zheng Liu, Hui Huang

## References

[1] Swyer GI. Male pseudohermaphroditism: a hitherto undescribed form. Brit Med J 1955;2:709–12. 13250193 10.1136/bmj.2.4941.709 PMC1980764

[2] Bayraktar Z. Potential autofertility in true hermaphrodites. J Matern Fetal Neonatal Med 2018;31:542–7. 28282768 10.1080/14767058.2017.1291619

[3] van Niekerk WA Retief AE. The gonads of human true hermaphrodites. Hum Genet 1981;58:117–22. 6895206 10.1007/BF00284158

[4] Irkilata HC Basal S Taslipinar A. Ovotesticular disorder of sex development with a prostatic gland and review of literature. Andrologia 2009;41:387–91. 19891638 10.1111/j.1439-0272.2009.00945.x

[5] Becker RE Akhavan A. Prophylactic bilateral gonadectomy for ovotesticular disorder of sex development in a patient with mosaic 45, X/46, X,idic(Y)q11.222 Karyotype. Urol Case Rep 2016;5:13–6. 26793590 10.1016/j.eucr.2015.12.003 PMC4719899

[6] Matsui F Shimada K Matsumoto F. Long-term outcome of ovotesticular disorder of sex development: a single center experience. Int J Urol 2011;18:231–6. 21255100 10.1111/j.1442-2042.2010.02700.x

[7] McCann-Crosby B Gunn S Smith EO. Association of immunohistochemical markers with premalignancy in gonadal dysgenesis. Int J Pediatr Endocrinol 2015;2015:14. 26089923 10.1186/s13633-015-0010-6 PMC4472165

[8] Watanabe S Aihara Y Kikuno A. A highly sensitive and specific chemiluminescent enzyme immunoassay for placental alkaline phosphatase in the cerebrospinal fluid of patients with intracranial germinomas. Pediatr Neurosurg 2012;48:141–5. 23429277 10.1159/000345632

[9] Gao Y Jiang J Liu Q. Clinicopathological and immunohistochemical features of primary central nervous system germ cell tumors: a 24-years experience. Int J Clin Exp Pathol 2014;7:6965–72. 25400782 PMC4230147

[10] Rodriguez-Buritica D. Overview of genetics of disorders of sexual development. Curr Opin Pediatr 2015;27:675–84. 26335769 10.1097/MOP.0000000000000275

[11] Arboleda VA Sandberg DE Vilain E. DSDs: genetics, underlying pathologies and psychosexual differentiation. Nat Rev Endocrinol 2014;10:603–15. 25091731 10.1038/nrendo.2014.130 PMC4441533

[12] Shabsovich D Tirado CA. Genes, chromosomes, and disorders of sex development: an update. J Assoc Genet Technol 2014;40:124–30. 26030029

[13] Ohnesorg T Vilain E Sinclair AH. The genetics of disorders of sex development in humans. Sex Dev 2014;8:262–72. 24504012 10.1159/000357956

[14] Arango NA Kobayashi A Wang Y. A mesenchymal perspective of Müllerian duct differentiation and regression in Amhr2-lacZ mice. Mol Reprod Dev 2008;75:1154–62. 18213646 10.1002/mrd.20858

